# Antibody microarray analysis of amniotic fluid proteomes in women with cervical insufficiency and short cervix, and their association with pregnancy latency length

**DOI:** 10.1371/journal.pone.0263586

**Published:** 2022-02-07

**Authors:** Subeen Hong, Kyo Hoon Park, Young Eun Lee, Ji Eun Lee, Yu Mi Kim, Eunwook Joo, Iseop Cho

**Affiliations:** 1 Department of Obstetrics and Gynecology, College of Medicine, The Catholic University of Korea, Seoul, Korea; 2 Department of Obstetrics and Gynecology, Seoul National University College of Medicine, Seoul National University Bundang Hospital, Seongnam, Korea; 3 Center for Theragnosis, Biomedical Research Division, Korea Institute of Science and Technology, Seoul, Korea; Universita degli Studi dell’Insubria, ITALY

## Abstract

**Introduction:**

This study aimed to investigate amniotic fluid (AF) proteins that were differentially expressed between patients with cervical insufficiency (CI) and asymptomatic short cervix (SCX, ≤ 25 mm), and whether these proteins could be predictive of spontaneous preterm birth (SPTB) in these patients.

**Method:**

This was a retrospective cohort study of 129 singleton pregnant women with CI (n = 80) or SCX (n = 49) at 17 to 26 weeks who underwent amniocentesis. An antibody microarray was used to perform comparative proteomic profiling of AF from matched CI (n = 20) and SCX (n = 20) pregnancies. In the total cohort, an ELISA validation study was performed for 15 candidate proteins of interest. Subgroup analyses of patients with CI and SCX were conducted to evaluate the association between the 15 proteins and SPTB at < 32 weeks of gestation.

**Results:**

Eighty-six proteins showed intergroup differences. ELISA validation confirmed significantly higher levels of AF EN-RAGE, IL-8, lipocalin-2, MMP-9, S100A8/A9, thrombospondin-2, and TNFR2 in patients with CI than in those with SCX. Multivariable analysis showed that increased AF levels of EN-RAGE, S100A8/A9, and uPA were independently associated with SPTB at < 32 weeks in patients with CI; whereas in patients with SCX, high AF levels of APRIL, EN-RAGE, LBP, and TNFR2 were independently associated with SPTB at < 32 weeks.

**Conclusions:**

Multiple AF proteins show altered expression in patients with CI compared with SCX controls. Moreover, several novel mediators involved in inflammation were identified as potential biomarkers for predicting SPTB after the diagnosis of CI and SCX. These results provide new insights into target-specific molecules for targeted therapies to prevent SPTB in patients with CI/SCX.

## Introduction

Cervical insufficiency (CI), defined as the inability of the uterine cervix to maintain a pregnancy in the absence of contractions, and asymptomatic short cervix (SCX), characterized by a cervix that is ≤ 25 mm in length by ultrasound measurement, in the mid-trimester remain significant obstetric problems affecting 0.1–1% of all pregnancies and 0.5% of asymptomatic singleton women at 16–23 weeks of gestation [1–3], respectively, and they represent important antecedents for spontaneous preterm birth (SPTB) [4]. Intra-amniotic inflammation and premature cervical changes are among the several events that contribute for the development of CI and asymptomatic SCX [4–6]. Moreover, cervical insufficiency/sufficiency is not a condition of “all-or-nothing;” instead, it is considered a continuum which can be assessed through transvaginal cervical length measurements [4, 7, 8]. Therefore, asymptomatic SCX in the mid-trimester may be, at least in part, associated with increased risk of CI and subsequent SPTB [4, 7]. To date, the proteins and mechanisms underlying such a continuum, and their contribution for subsequent SPTB, have not been fully elucidated. Hence, better understanding of such molecular and cellular mechanisms may provide useful information for developing effective targeted prevention and treatment strategies for CI and asymptomatic SCX.

Previous reports have identified potential proteins involved in the underlying mechanisms of cervical competence continuum and subsequent SPTB, including interleukin (IL)-6 in amniotic fluid (AF), complement components 3a and 5a in maternal blood, as well as IL-6, tissue inhibitor of metalloproteinases-1, Dickkopf (DKK) 3, and vitamin D binding protein in cervicovaginal fluid [9–13]. However, most of these studies have only addressed specific target proteins, thus not reflecting all proteins having a role in the complex network of mechanisms involved in the continuum of cervical competence. In contrast to conventional, single analyte approaches for protein analysis (such as enzyme-linked immunosorbent assays [ELISA]), antibody microarray is a high-throughput technique that allows simultaneous identification of hundreds of proteins related to a specific condition; thus, it has proposed as an useful screening tool for biomarker discovery and for investigating signal pathways [14].

The present study aimed to investigate the proteins and their related signaling pathways that were differentially expressed in AF between women with CI and SCX (≤ 25 mm) using an antibody microarray. Moreover, the identified deregulated proteins were also evaluated regarding their predictive potential for SPTB in patients with CI and SCX.

## Materials and methods

### Study design and participants

This retrospective cohort study involved 129 asymptomatic singleton pregnant women diagnosed with SCX (n = 49) or CI (n = 80) at 17 and 26 weeks of gestation who were admitted to the high-risk maternity unit of the Seoul National University Bundang Hospital (Seongnamsi, Republic of Korea) between September 2004 and July 2018. Women who underwent transabdominal amniocentesis to assess AF for possible subclinical infection/inflammation [4, 15–18] and who delivered a live fetus were included in the study. Women with 1) preterm premature rupture of the membranes (PPROM), preterm contraction, or preterm labor (PTL) at diagnosis; 2) multifetal pregnancy; 3) prophylactic cerclage during early pregnancy; 4) evidence of clinical chorioamnionitis at admission; and/or 5) major congenital anomalies were excluded from the study. SCX was defined as a transvaginal sonographic cervical length of ≤ 25 mm, measured using the standard technique as previously described [19]. CI was defined as a painless dilation of the cervix in the mid-trimester ≥ 1 cm with fetal membranes visual at or beyond the external os, as determined by a sterile speculum examination, without contractions or labor. Gestational age was determined based on menstrual dating along with an ultrasound scan performed in the first or early second trimester. The ethics committee of the Seoul National University Bundang Hospital approved the study (project number B-1105/128-102). Written informed consent was obtained from all patients for the amniocentesis procedure, as well as for the use of the AF samples and clinical data for research purposes.

A nested case-control approach was used for the protein discovery study, comprising 20 patients with CI (case group) and 20 patients with SCX (control group). CI cases were randomly chosen from the list of eligible patients with CI (n = 80) using a random sequence generator. For each case patient, control patients were selected by matching gestational age at amniocentesis, parity, maternal age, and years of sampling.

#### Biological sample collection and processing

Collection, preparation, and storage of AF samples, and the identification of microorganisms were performed as previously reported [20]. Briefly, at the time of admission, transabdominal ultrasound-guided amniocentesis was performed using a 22-gauge spinal needle. Presence of microorganisms, such as aerobic/anaerobic bacteria, fungi, and genital mycoplasmas (*Ureaplasma urealyticum* and *Mycoplasma hominis*), in AF samples was determined by conventional culture-based methods, as previously described [20]. The remaining AF was centrifuged for 10 min at 1,500 × *g*, and the supernatant was aliquoted and stored at −70°C until use.

#### Membrane-based human antibody array

AF proteome profiles of SCX control and CI case groups (n = 20 in each group) were determined using a Human Antibody Array Kit (AAH-BLM-1B-2; RayBiotech, Norcross, GA, USA) comprising 507 different immune-regulatory proteins, including cytokines, chemokines, matrix metalloproteases, adhesion molecules, growth and angiogenic factors, and adipokines (described in detail in previous studies [21, 22]). The detailed detection process of the protein antibody microarray and image analyses are provided in S3 File. Briefly, equal amounts (25 μg) of protein from the individual AF samples were pooled into case (CI, n = 20) and control (SCX, n = 20) samples, up to a total of 500 μg per group, for use in the high-throughput protein microarray. The pooled AF samples were analyzed in duplicate according to the manufacturer’s protocol. To identify target molecules showing significant signal intensity differences between CI cases and SCX controls, protein spots were selected based on the following criteria: (1) fold change (FC) of ≥ 1.3 or ≤ 0.77 for up- or downregulated proteins, respectively, based on the chemiluminescence image analysis, and (2) visible to the naked eye.

#### ELISA validation

To verify the results of the antibody microarray data, the AF levels of the proliferation-inducing ligand (APRIL), DKK3, endostatin, advanced glycation end products binding protein (EN-RAGE), insulin-like growth factor-binding protein (IGFBP)-2, IL-8, lipocalin-2, lipopolysaccharide binding protein (LBP), matrix metalloproteinase (MMP)-2, MMP-9, S100 calcium-binding protein A8/A9 complex (S100A8/A9), secreted protein acidic and rich in cysteine (SPARC), thrombospondin-2 (TSP2), tumor necrosis factor receptor 2 (TNFR2), and urokinase-type plasminogen activator (uPA) were evaluated in the final cohort of 129 subjects using ELISA kits (DuoSet ELISA; R&D Systems, Minneapolis, MN, USA), according to the manufacturer’s instructions. The ranges for each protein standard curves and dilution ratios are provided in detail in S3 File. The intra- and inter-assay coefficients of variation (CVs) were < 10% for all analyzed proteins, except for APRIL and DKK3, for which the inter-assay CVs were 10.5% and 12.8%, respectively. These molecules were selected for the validation study because ⅰ) they revealed a high expression FC; and ⅱ) few information was available to date concerning their expression change in the AF of women with CI/SCX. Ubiquitin-1 and vascular endothelial growth factor were also evaluated in AF samples by dilution linearity and spike/recovery testing, but the results showed poor assay performance; therefore, quantification of these proteins was not performed.

#### Management of CI and asymptomatic SCX, and definitions of various factors

Management of CI or asymptomatic SCX was performed as previously reported [23, 24] and is also described in S3 File. Briefly, decisions regarding cervical cerclage, bed rest, and use of antibiotics, progesterone, and tocolytic agents, were made based on the discretion of the attending physician. For maturation of fetal lungs, antenatal corticosteroids were administered between 24 and 34 weeks of gestation. The diagnosis of acute histologic chorioamnionitis and clinical chorioamnionitis, and the techniques to measure cervical length using transvaginal sonography have been previously described in detail [19, 20, 25, 26], and a detailed description is provided in S3 File. SPTB was defined as preterm birth caused by PTL, PPROM, or the development of clinical chorioamnionitis.

#### Ingenuity pathway analysis

The Ingenuity Pathway Analysis (IPA) bioinformatics tool (Version 49932394; QIAGEN, Hilden, Germany) was used to determine the functional annotation and molecular pathways that were associated with the differentially expressed proteins (DEPs) identified between CI and SCX samples. UniProt accession numbers of the DEPs, and their corresponding log_2_ ratios, were uploaded into the IPA software online.

#### Statistical methods

Comparisons between groups were performed using the Student’s *t*-test or Mann-Whitney *U* test for continuous data, and the χ^2^ test or Fisher’s exact test for categorical data, as appropriate. Logistic regression analysis was performed to assess the independent association between the AF levels of each protein and the outcome measure, after adjusting for baseline clinical variables with a *P*-value < 0.05 in the univariate analysis. Receiver operating characteristic (ROC) curves were generated to determine the optimal cutoff and predictive values of each studied protein in the AF for the outcome measure, and the area under the curves (AUCs) were compared as previously described [27]. Correlations between non-normally distributed continuous variables were examined using the Spearman’s rank correlation test. Data analysis was conducted using SPSS version 25.0 (IBM Corp., Armonk, NY, USA). The significance level was set at 0.05 (two-tailed) for all statistical analyses.

## Results

### Discovery phase

The baseline characteristics of the exploratory cohorts used for the antibody microarray analysis are presented in S1 Table. Owing to matching, CI cases and SCX controls were similar for gestational age at amniocentesis, parity, and maternal age.

**Fig 1:**
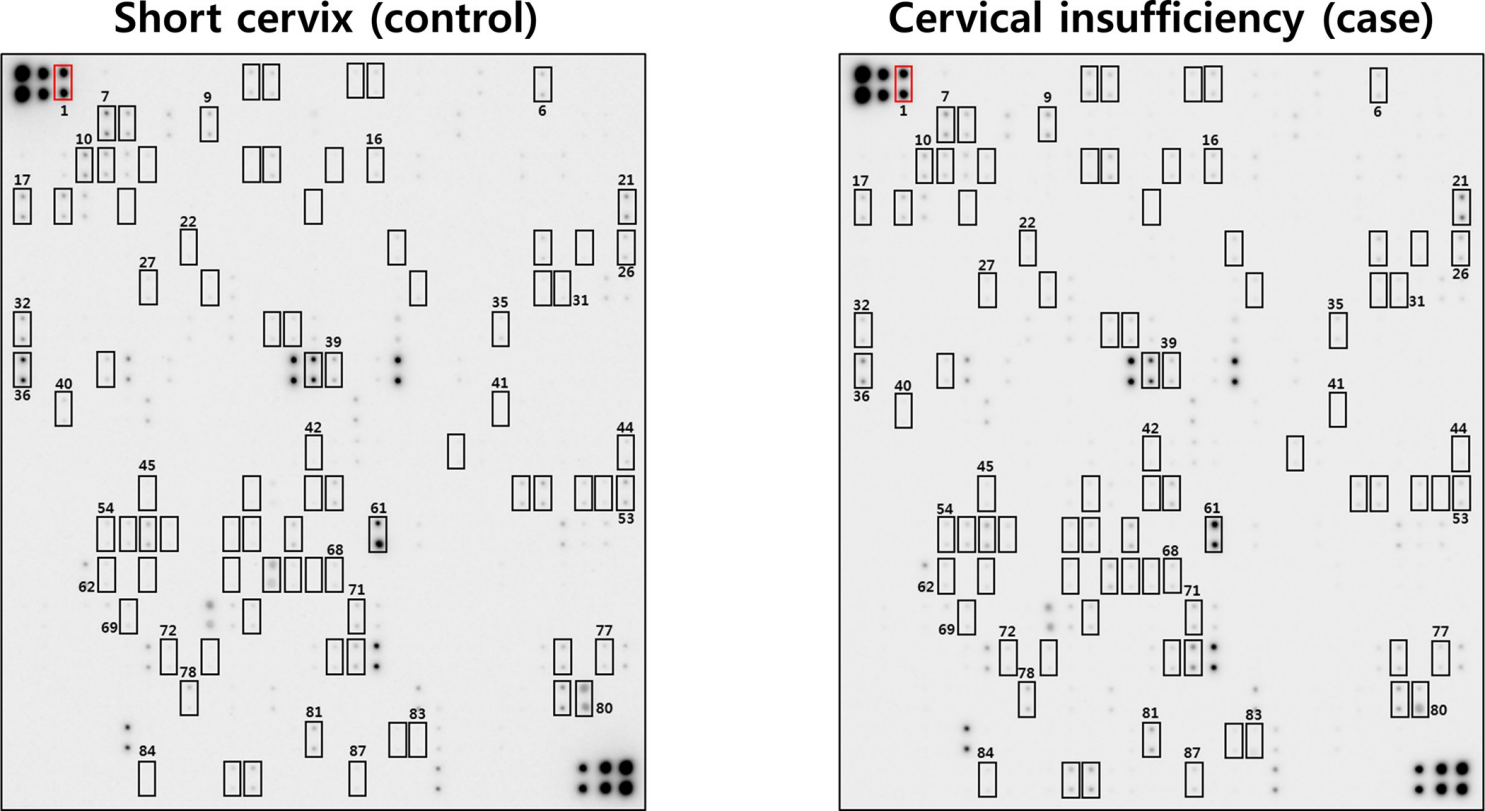
Expression profile of 507 proteins in the amniotic fluid (AF) of patients with cervical insufficiency (CI, case subjects) versus short cervix (SCX, control subjects). Pooled AF samples from each group (20 samples per group, matched for gestational age) were assayed using a human antibody array kit. Fold change cutoff values of ≥ 1.3 or ≤ 0.77 for upregulated or downregulated proteins, respectively, revealed 86 differentially expressed proteins (indicated in rectangles) in AF samples from patients with CI relative to SCX controls. Number 1 indicates the positive controls.

Using the spot selection criteria (described in Materials and Methods), 86 out of 507 human proteins included in the membrane-based microarray were found to be differentially expressed among AF samples from patients with SCX and those with CI (Fig 1). Overall, 60 proteins were upregulated and 26 were downregulated in CI compared with SCX (S2 Table). These 86 proteins were further analyzed using IPA to assess their canonical pathways and biological functions (S3 Table). Five main pathways were identified, including ‘granulocyte/agranulocyte adhesion and diapedesis,’ ‘hepatic fibrosis/hepatic stellate cell activation,’ and ‘cardiac hypertrophy signaling.’ Moreover, IPA also identified ‘connective tissue disorders,’ ‘inflammatory disease,’ ‘organismal injury and abnormalities,’ ‘skeletal and muscular disorders,’ and ‘immunological disease’ as top disease and disorder functions related to these 86 DEPs.

#### ELISA validation phase

Fifteen candidate proteins identified by the antibody microarray—APRIL, DKK3, endostatin, EN-RAGE, IGFBP-2, IL-8, LBP, lipocalin-2, MMP-2, MMP-9, S100A8/A9, SPARC, TSP2, TNFR2, and uPA—were further validated by quantitative ELISA in 129 individual samples (80 CI and 49 SCX samples). The median AF levels of EN-RAGE, IL-8, lipocalin-2, MMP-9, S100A8/A9, TSP2, and TNFR2 were significantly higher in patients with CI than in patients with SCX (Table 1). However, the AF levels of APRIL, DKK3, endostatin, IGFBP-2, LBP, MMP-2, SPARC, and uPA, as well as demographic factors did not significantly differ between the two groups (Table 1).

**Table 1:** Demographic and clinical characteristics, and quantification of the selected candidate biomarkers of interest in women with cervical insufficiency or a short cervix included in the study.

Characteristics	Disease entity		*P*-value
	Cervical insufficiency (n = 80)	Short cervix (n = 49)	
Age (years)	32.4 ± 3.3	32.2 ± 3.5	0.766
Nulliparity	51.3% (41/80)	57.1% (28/49)	0.517
Gestational age at amniocentesis (weeks)	21.7 ± 2.3	22.0 ± 2.1	0.523
Cerclage placement	66.3% (53/80)	75.5% (37/49)	0.268
Vaginal progesterone therapy	32.5% (26/80)	61.2% (30/49)	**0.001**
Positive AF cultures	8.8% (7/80)	8.2% (4/49)	1.000
Cervical dilatation (cm)	2.0 (1.0–8.0)		
≥ 3 cm	47.5% (38/80)		
< 3 cm	52.5% (42/80)		
Cervical length by ultrasound (mm)		9.6 ± 4.5	
AF APRIL (ng/mL)	0.75 ± 0.52	0.79 ± 1.16	0.280
AF DKK3 (ng/mL)	229.23 ± 84.39	230.03 ± 62.51	0.687
AF endostatin (ng/mL)	62.72 ± 20.57	59.32 ± 19.89	0.348
AF EN-RAGE (ng/mL)	26.92 ± 19.72	11.40 ± 15.14	**<0.001**
AF IGFBP-2 (ng/mL)	1362.68 ± 494.45	1190.80 ± 483.11	0.060
AF IL-8 (ng/mL)	6.54 ± 6.67	1.89 ± 4.53	**<0.001**
AF LBP (ng/mL)	652.24 ± 437.78	566.05 ± 412.27	0.268
AF lipocalin-2 (ng/mL)	1260.10 ± 753.55	625.79 ± 574.49	**<0.001**
AF MMP-2 (ng/mL)	105.97 ± 31.97	102.82 ± 28.24	0.483
AF MMP-9 (ng/mL)	5.75 ± 5.84	2.07 ± 4.23	**<0.001**
AF S100 A8/A9 (ng/mL)	2004.38 ± 1012.29	1133.56 ± 909.59	**<0.001**
AF SPARC (ng/mL)	906.79 ± 488.18	915.42 ± 462.89	0.985
AF TSP2 (ng/mL)	136.12 ± 70.53	89.92 ± 40.51	**<0.001**
AF TNFR2 (ng/mL)	15.25 ± 9.91	11.48 ± 8.38	**<0.001**
AF uPA (ng/mL)	0.32 ± 0.29	0.62 ± 2.27	0.184
Gestational age at delivery (weeks)	29.6 ± 7.2	34.7 ± 5.7	**<0.001**

AF, amniotic fluid; APRIL, a proliferation-inducing ligand; DKK3, dickkopf-related protein 3; EN-RAGE (S100A12), extracellular newly identified receptor for advanced glycation end products binding protein; IGFBP, insulin-like growth factor-binding protein; IL, interleukin; LBP, lipopolysaccharide binding protein; MMP, matrix metalloproteinase; S100 A8/A9, S100 calcium-binding protein A8/A9 complex; SPARC, secreted protein acidic and rich in cysteine; TSP2, thrombospondin-2; TNFR2, tumor necrosis factor receptor 2; uPA, urokinase-type plasminogen activator.

Values are presented as mean ± standard deviation, median (range), or % (n/N).

Significant findings (*P* < 0.05) are indicated in bold fonts.

#### Subgroup analysis of patients with CI

A subgroup analysis was conducted according to the disease entity to evaluate the association between the 15 protein markers in AF and SPTB at < 32 weeks of gestation. In the subgroup analysis of patients with CI, the median AF levels of EN-RAGE, IL-8, lipocalin-2, MMP-9, S100A8/A9, and uPA were significantly higher in women with CI who had SPTB at < 32 weeks than in those who delivered at ≥ 32 weeks (Table 2). Furthermore, multivariate analysis revealed that elevated AF levels of EN-RAGE, S100A8/A9, and uPA (but not IL-8, lipocalin-2, or MMP-9) were significantly associated with SPTB at < 32 weeks after adjusting for cervical dilatation, cerclage placement, and vaginal progesterone therapy (Table 3).

**Table 2:** Demographic and clinical characteristics, and quantification of the selected candidate biomarkers of interest in women with cervical insufficiency stratified according to the occurrence of spontaneous preterm birth.

Characteristics	Delivery < 32 weeks (n = 44)	Delivery ≥ 32 weeks (n = 36)	*P*-value
Age (years)	32.2 ± 3.5	32.6 ± 3.1	0.323
Nulliparity	56.8% (25/44)	44.4% (16/36)	0.225
Gestational age at amniocentesis (weeks)	21.7 ± 2.5	21.6 ± 1.9	0.768
Cervical dilatation (cm)	3.0 (1.0–8.0)	2.0 (1.0–8.0)	**0.006**
≥ 3 cm	59.1% (26/44)	33.3% (12/36)	**0.022**
< 3 cm	40.9% (18/44)	66.7% (24/36)	
Cerclage placement	47.7% (21/44)	88.9% (32/36)	**<0.001**
Vaginal progesterone therapy	13.6% (6/44)	55.6% (20/36)	**<0.001**
Positive AF cultures	15.9% (7/44)	0.0% (0/36)	**0.015**
Use of antibiotics	95.5% (42/44)	97.2% (35/36)	0.679
Use of antenatal steroid	40.9% (18/44)	22.2% (8/36)	0.076
Use of tocolytics	52.3% (23/44)	33.3% (12/36)	0.089
AF APRIL (ng/mL)	0.82 ± 0.59	0.67 ± 0.42	0.451
AF DKK3 (ng/mL)	224.89 ± 95.93	234.67 ± 68.67	0.292
AF endostatin (ng/mL)	63.10 ± 20.26	62.24 ± 21.25	0.767
AF EN-RAGE (ng/mL)	33.76 ± 17.92	18.56 ± 18.79	**<0.001**
AF IGFBP-2 (ng/mL)	1361.85 ± 532.19	1363.70 ± 451.51	0.884
AF IL-8 (ng/mL)	7.78 ± 6.59	5.02 ± 6.54	**0.008**
AF LBP (ng/mL)	735.22 ± 504.48	550.81 ± 317.29	0.173
AF lipocalin-2 (ng/mL)	1461.18 ± 774.17	1014.33 ± 657.56	**0.006**
AF MMP-2 (ng/mL)	107.20 ± 37.77	104.46 ± 23.44	0.843
AF MMP-9 (ng/mL)	7.02 ± 6.68	4.19 ± 4.21	**0.044**
AF S100 A8/A9 (ng/mL)	2417.20 ± 862.09	1499.82 ± 961.15	**<0.001**
AF SPARC (ng/mL)	852.89 ± 552.11	972.69 ± 394.07	0.119
AF TSP2 (ng/mL)	140.10 ± 79.81	131.26 ± 57.97	0.832
AF TNFR2 (ng/mL)	17.43 ± 11.80	12.58 ± 6.11	0.072
AF uPA (ng/mL)	0.39 ± 0.36	0.24 ± 0.13	**0.024**
Gestational age at delivery (weeks)	23.8 ± 3.6	36.9 ± 2.1	**<0.001**

AF, amniotic fluid; APRIL, a proliferation-inducing ligand; DKK3, dickkopf-related protein 3; EN-RAGE (S100A12), extracellular newly identified receptor for advanced glycation end products binding protein; IGFBP, insulin-like growth factor-binding protein; IL, interleukin; LBP, lipopolysaccharide binding protein; MMP, matrix metalloproteinase; S100 A8/A9, S100 calcium-binding protein A8/A9 complex; SPARC, secreted protein acidic and rich in cysteine; TSP2, thrombospondin-2; TNFR2, tumor necrosis factor receptor 2; uPA, urokinase-type plasminogen activator.

Values are presented as mean ± standard deviation, median (range), or % (n).

Significant findings (*P* < 0.05) are indicated in bold fonts.

**Table 3:** Multivariable logistic regression model showing the unadjusted and adjusted odds ratios of an association between various proteins in amniotic fluid and spontaneous preterm birth at < 32 weeks in women with cervical insufficiency and a short cervix.

Variables	Odds ratio (95% confidence interval)		*P*-value^§^
	Unadjusted	Adjusted	
**Cervical insufficiency** ^†^			
AF EN-RAGE (ng/mL)	1.044 (1.018–1.070)	1.037(1.005–1.070)	**0.022**
AF IL-8 (ng/mL)	1.069 (0.994–1.150)	1.055 (0.958–1.161)	0.275
AF lipocalin-2 (ng/mL)	1.001 (1.000–1.002)	1.001 (1.000–1.002)	0.055
AF MMP-9 (ng/mL)	1.099 (1.005–1.202)	1.084 (0.968–1.214)	0.162
AF S100 A8/A9 (ng/mL)	1.001 (1.000–1.002)	1.001 (1.000–1.002)	**0.006**
AF uPA (ng/mL)	27.084 (1.515–484.117)	365.087 (2.378–56055.036	**0.022**
**A short cervix** ^‡^			
AF APRIL (ng/mL)	14.552 (2.166–97.774)	13.644 (1.639–113.541)	**0.016**
AF EN-RAGE (ng/mL)	1.058 (1.014–1.104)	1.089 (1.025–1.157)	**0.006**
AF LBP (ng/mL)	1.004 (1.000–1.007)	1.005 (1.001–1.009)	**0.014**
AF TNFR2 (ng/mL)	1.128 (1.002–1.271)	1.170 (1.028–1.332)	**0.018**

AF, amniotic fluid; EN-RAGE (S100A12), extracellular newly identified receptor for advanced glycation end products binding protein; IL, interleukin; MMP, matrix metalloproteinase; S100 A8/A9, S100 calcium-binding protein A8/A9 complex; uPA, urokinase-type plasminogen activator; APRIL, a proliferation-inducing ligand; LBP, lipopolysaccharide binding protein; TNFR2, tumor necrosis factor receptor 2.

^†^ Adjustment for cervical dilatation, cerclage placement, and vaginal progesterone therapy.

^‡^ Adjustment for gestational age at sampling.

§ For the adjusted odds ratio.

Significant findings (*P* < 0.05) are indicated in bold fonts.

**Fig 2:**
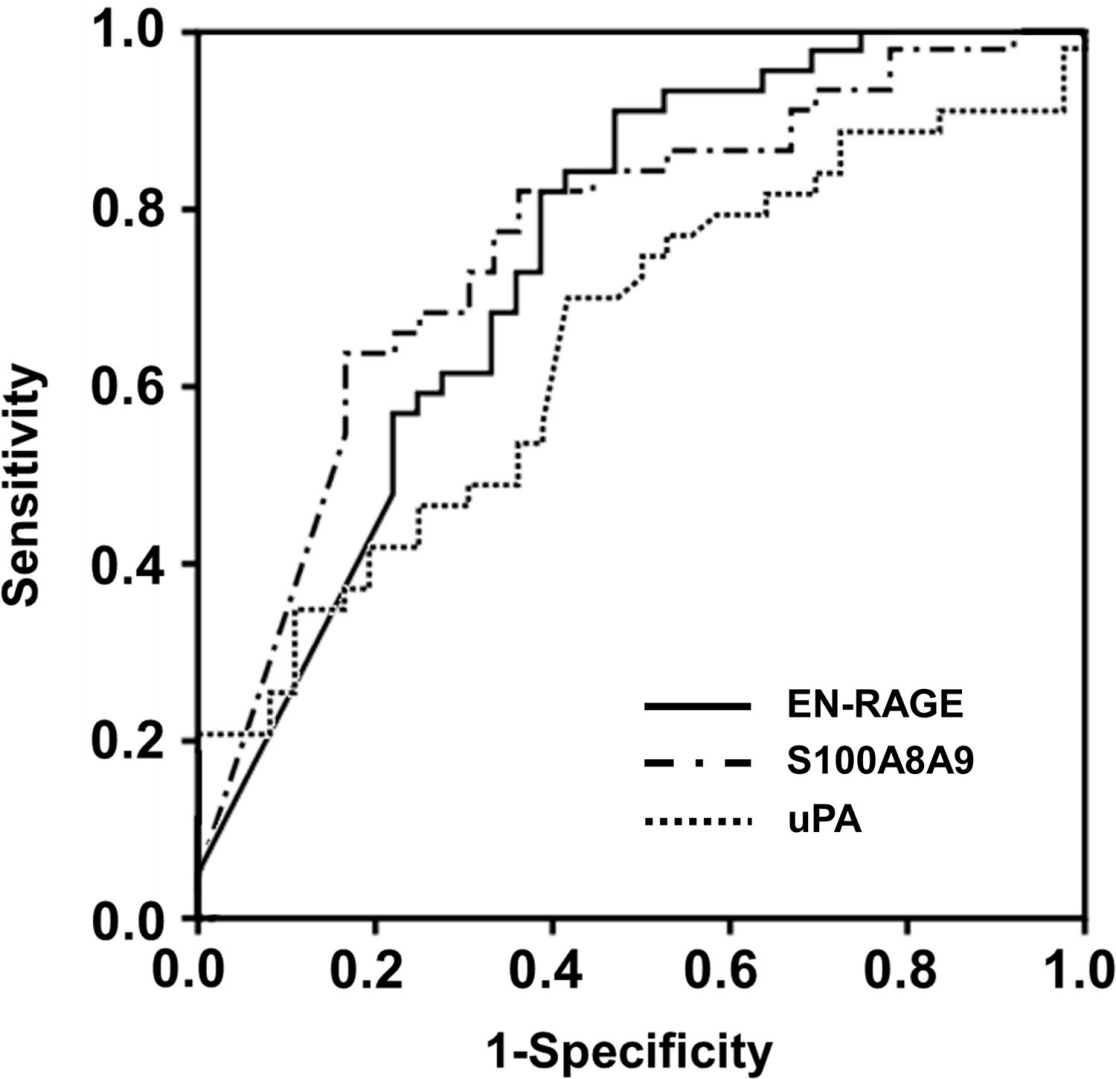
Receiver operating characteristic (ROC) curves of amniotic fluid (AF) EN-RAGE, S100A8/A9, and uPA at predicting spontaneous preterm birth (SPTB) at < 32 weeks of gestation (AF EN-RAGE: area under the curve [AUC] = 0.744, SE = 0.057; AF S100A8/A9: AUC = 0.763, SE = 0.054; and AF uPA: AUC = 0.649, SE = 0.062). Differences among the AUCs of AF EN-RAGE, S100A8/A9, and uPA were not significant (all variables: *P* = 0.09–0.66). EN-RAGE, extracellular newly identified receptor for advanced glycation end products binding protein; S100A8/A9, S100 calcium binding protein A8/A9 complex; uPA, urokinase-type plasminogen activator.

AUC values of AF EN-RAGE, S100A8/A9, and uPA for the prediction of SPTB at < 32 weeks of gestation were 0.744, 0.763, 0.755, and 0.649, respectively (S4 Table and Fig 2), not showing significant differences among them (all variables: *P* = 0.09–0.66). Moreover, the concentration of the AF proteins was found to be significantly associated with SPTB at < 32 weeks of gestation (EN-RAGE, S100A8/A9, and uPA), showing a significant correlation with each other (all proteins, r = 0.35–0.84, *P* < 0.005).

#### Subgroup analysis of patients with a SCX

**Fig 3:**
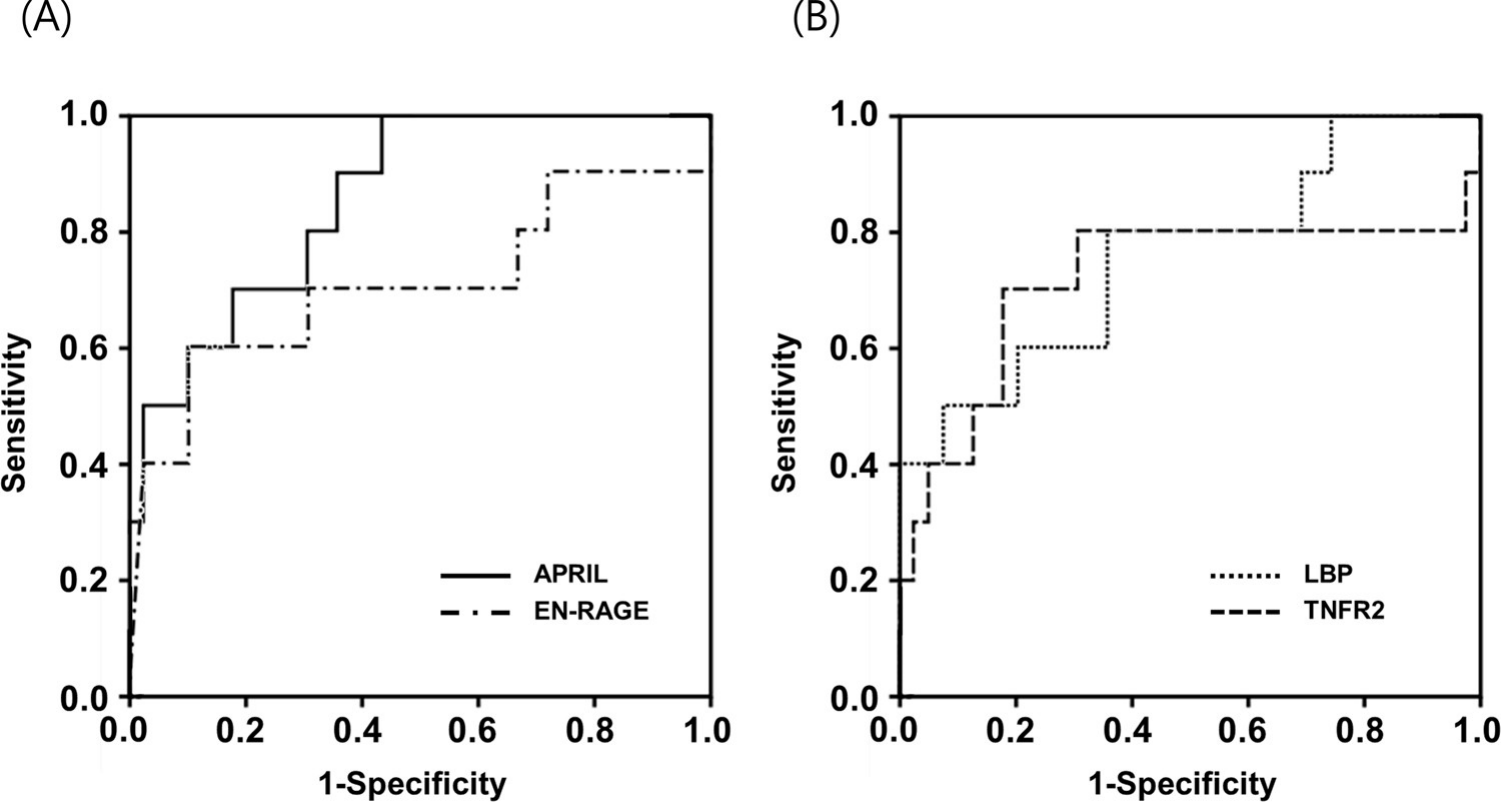
(A) Receiver operating characteristic (ROC) curves of amniotic fluid (AF) APRIL and EN-RAGE at predicting spontaneous preterm birth (SPTB) at < 32 weeks of gestation (AF APRIL: area under the curve [AUC] = 0.856, SE = 0.060; and AF EN-RAGE: AUC = 0.705, SE = 0.114). (B) ROC curves of AF LBP and TNFR2 at predicting SPTB at < 32 weeks of gestation (AF LBP: AUC = 0.756, SE = 0.093; and AF TNFR2: AUC = 0.715, SE = 0.118). Differences among the AUCs of AF APRIL, EN-RAGE, LBP, and TNFR2 were not significant (all variables: *P* = 0.09–0.73). APRIL, a proliferation-inducing ligand; EN-RAGE, extracellular newly identified receptor for advanced glycation end products binding protein; LBP, lipopolysaccharide binding protein; TNFR2, tumor necrosis factor receptor 2.

Univariate analyses of SCX patients showed that elevated AF levels of APRIL, EN-RAGE, LBP, and TNFR2 were significantly associated with SPTB at < 32 weeks (Table 4). Moreover, these associations remained significant in multivariable analysis, even after adjusting for gestational age at sampling (Table 3). In the subgroup of women with SCX, the AUCs for the prediction of SPTB at < 32 weeks were 0.856 for AF APRIL, 0.705 for AF EN-RAGE, 0.756 for AF LBP, and 0.715 for AF TNFR2 (S5 Table and Fig 3), which were not statistically significantly different (all variables: *P* = 0.09–0.73). The AF levels of APRIL, EN-RAGE, LBP, and TNFR2 were significantly correlated with each other (all variables, r = 0.209–0.592, *P* < 0.01), except for APRIL and EN-RAGE (r = 0.209, *P* = 0.150), and APRIL and LBP (r = 0.233, *P* = 0.107).

**Table 4:** Demographic and clinical characteristics, and quantification of the selected candidate biomarkers of interest in women with a short cervix stratified according to the occurrence of spontaneous preterm birth.

Characteristics	Delivery < 32 weeks (n = 10)	Delivery ≥ 32 weeks (n = 39)	*P*-value
Age (years)	30.7 ± 2.8	32.6 ± 3.6	0.053
Nulliparity	60.0% (6/10)	56.4% (22/39)	0.838
Gestational age at amniocentesis (weeks)	20.6 ± 1.8	22.4 ± 2.1	**0.020**
Cerclage placement	70.0% (7/10)	76.9% (30/39)	0.650
Vaginal progesterone therapy	70.0% (7/10)	58.9% (23/39)	0.523
Positive AF cultures	10.0% (1/10)	7.7% (3/39)	1.000
Use of antibiotics	100.0% (10/10)	79.5% (31/39)	0.117
Use of antenatal steroid	40.0% (4/10)	23.7% (9/38)	0.425
Use of tocolytics	60.0% (6/10)	33.3% (13/39)	0.123
Cervical length by ultrasound (mm)	10.0 ± 6.0	9.5 ± 4.1	0.775
AF APRIL (ng/mL)	1.87 ± 2.25	0.52 ± 0.33	**0.001**
AF DKK3 (ng/mL)	258.66 ± 82.39	222.70 ± 55.29	0.297
AF endostatin (ng/mL)	68.67 ± 20.66	59.95 ± 19.24	0.102
AF EN-RAGE (ng/mL)	23.91 ± 22.71	8.19 ± 10.75	**0.047**
AF IGFBP-2 (ng/mL)	1038.15 ± 501.38	1229.93 ± 477.04	0.229
AF IL-8 (ng/mL)	6.08 ± 8.97	0.81 ± 1.01	0.253
AF LBP (ng/mL)	997.32 ± 692.48	455.47 ± 199.06	**0.013**
AF lipocalin-2 (ng/mL)	1002.19 ± 936.46	529.27 ± 402.93	0.224
AF MMP-2 (ng/mL)	112.49 ± 42.72	100.34 ± 23.33	0.535
AF MMP-9 (ng/mL)	4.97 ± 8.04	1.33 ± 2.13	0.359
AF S100 A8/A9 (ng/mL)	1341.72 ± 1172.15	1080.18 ± 839.84	0.673
AF SPARC (ng/mL)	1010.57 ± 762.49	891.03 ± 360.49	0.728
AF TSP2 (ng/mL)	100.70 ± 53.74	87.16 ± 36.76	0.862
AF TNFR2 (ng/mL)	18.49 ± 15.89	9.68 ± 3.56	**0.037**
AF uPA (ng/mL)	2.13 ± 4.91	0.23 ± 0.15	0.244
Gestational age at delivery (weeks)	24.7 ± 4.6	37.2 ± 1.9	**<0.001**

AF, amniotic fluid; APRIL, a proliferation-inducing ligand; DKK3, Dickkopf-related protein 3; **EN-RAGE** (S100A12), extracellular newly identified receptor for advanced glycation end products binding protein; IGFBP, insulin-like growth factor-binding protein; IL, interleukin; LBP, lipopolysaccharide binding protein; MMP, matrix metalloproteinase; S100 A8/A9, S100 calcium-binding protein A8/A9 complex; SPARC, secreted protein acidic and rich in cysteine; TSP2, thrombospondin-2; TNFR2, tumor necrosis factor receptor 2; uPA, urokinase-type plasminogen activator.

Values are presented as mean ± standard deviation or % (n/N).

Significant findings (*P* < 0.05) are indicated in bold fonts.

## Discussion

The main findings of this study were the following: (ⅰ) using protein–antibody microarray analysis, seven novel proteins (i.e., EN-RAGE, IL-8, lipocalin-2, MMP-9, S100A8/A9, TSP2, and TNFR2) and their specific signaling pathways, most of which are involved in inflammation and extracellular matrix remodeling, were identified as being more significantly expressed in AF samples of CI cases than in SCX controls; (ⅱ) increased AF levels of EN-RAGE, S100A8/A9, and uPA are independently associated with SPTB at < 32 weeks in patients with CI; and (ⅲ) in patients with a SCX, high AF levels of APRIL, EN-RAGE, LBP, and TNFR2 are independently associated with SPTB at < 32 weeks. These results provide new insights into specific biomarkers that could be valuable for targeted strategies to prevent SPTB in patients with CI/SCX and help to understand the underlying mechanisms of CI based on the hypothesis that SCX and CI might represent a continuum of risk for the development of SPTB.

Among the biomarkers analyzed, only the AF levels of EN-RAGE were found to be independently predictive of SPTB at < 32 weeks in both patients with CI and those with SCX, with relatively good accuracy. Moreover, this protein level is significantly higher in the AF from patients with CI than in that from those with SCX. Taken together, these data suggest that in the asymptomatic context, EN-RAGE may play an important role in the process of cervical ripening, dilation, and preterm parturition, but it may also have the potential to be clinically used to determine whether preterm birth may occur. EN-RAGE (also known as calgranulin C or S100A12) is mainly involved in the regulation of inflammatory processes and immune responses by binding with RAGE [28]. Thus, EN-RAGE has been proposed as a useful biomarker for inflammation-related disease [28]. In particular, Buhimschi *et al*. used proteomic fingerprint technology to show that EN-RAGE expression is upregulated in the AF during microbial invasion of the amniotic cavity (MIAC), intra-amniotic inflammation (IAI), and histologic chorioamnionitis (HCA) in pregnancies complicated by PTL or PPROM [29–31]. These data are in agreement with the herein reported ELISA-based results of AF EN-RAGE, along with its reported associations with SPTB risk in CI/SCX [12, 32].

The present study shows that elevated levels of EN-RAGE, S100A8/A9, and uPA in AF could independently predict SPTB at < 32.0 weeks in women with CI. S100A8/A9 (also known as calprotectin), which is in the form of a heterodimer, is primarily released from neutrophils, monocytes, and macrophages and plays an important role in the regulation of antimicrobial and inflammatory responses, and in the induction of cytokine secretion [33]. Previous studies have shown that S100A8/A9 expression is upregulated in the AF or maternal serum of pregnancies complicated by SPTB, HCA, IAI, and preeclampsia [22, 34–36]. Herein, S100A8/A9 levels in the AF of women with CI were found to be significantly associated with SPTB risk as well. uPA (also known as urokinase) is a potent 50 kDa serine protease that catalyzes the conversion of plasminogen to plasmin, contributing to the degradation of the extracellular matrix and thrombolysis [37]. Data from previous studies have shown that TNF-α, which is involved in preterm parturition, stimulates the biosynthesis of uPA and MMPs in cultured human chorionic cells, and that uPa is strongly expressed in the fetal membranes collected after spontaneous labor at term [38, 39]. These studies support the present findings on the association between uPA and SPTB risk in CI women, as both term and preterm parturition involve common phenotypic changes, including cervical remodeling and dilatation [40].

Assessment of women with SCX revealed that AF levels of APRIL, EN-RAGE, LBP, and TNFR2 were significantly and independently associated with SPTB at < 32 weeks. These results agree with those of previous studies showing that AF inflammatory score of ≥ 8 (, which is assigned using 14 inflammatory mediators in AF) and AF monocyte chemoattractant protein-1 were predictive of delivery at < 34 weeks in patients with mid-trimester SCX [41, 42]. APRIL (also known as TNFSF13) is a protein that binds to B-cell maturation antigen and plays an important role in the immune system and apoptosis [43]. This protein is mainly expressed in immune cells, such as monocytes, macrophages, and T cells, as well as in the placenta, including in villous cytotrophoblast cells [44]. To date, no studies have reported the association between APRIL in AF with SPTB, MIAC, or IAI. Herein, AF APRIL was found to be strongly independently associated with SPTB at < 32 weeks in SCX women. This is consistent with data published from a recent transcriptomic study on human normal term labor, demonstrating that TNFRSF13B, which has been shown to interact with APRIL [45], may play an important role in regulating the labor transcriptome in the choriodecidua [46]. LBP is a protein that binds to lipopolysaccharide (LPS) and plays a critical role in the innate immune response to both gram-negative and gram-positive microbes [47]. Moreover, this protein initiates a process that triggers the release of cytokines, with the attachment of the LPS-LBP complex to CD14 [47, 48]. Previous studies revealed that the levels of LBP were significantly higher in the AF of PTL pregnancies complicated by SPTB, MIAC, or IAI [49, 50], which is in line with the herein reported association of LBP with SPTB at < 32 weeks in the context of SCX. TNFR2 is a receptor for TNF-α, which is known to be a potent pleiotropic promoter of inflammation [51]. TNFR2 is mostly expressed in immune cells and has proinflammatory effects, but also elicits strong anti-inflammatory and cell-protective activities [51, 52]. A previous study showed that TNFR2 in AF has been associated with SPTB in women with PTL, irrespective of the presence of MIAC and cervical ripening at term parturition [53], which agrees with the newly found association between TNFR2 and SPTB in women with SCX.

The levels of several inflammatory (EN-RAGE, IL-8, lipocalin-2, S100-A8/A9, and TNFR2) and extracellular matrix-related (MMP-9 and TSP2) proteins were found to be significantly higher in the AF of patients with CI than in that of patients with SCX. These results are similar to those of previous studies by Weiner *et al*. and Son *et al*., who demonstrated that the AF levels of various cytokines and MMP-8, as well as the Mass-Restricted score (that was associated with intra-uterine inflammation), were significantly increased in patients with CI compared with healthy pregnant women (genetic amniocentesis) [54, 55]. We acknowledge that comparing the results of the current study involving women with CI and SXC directly to those of the aforementioned studies involving women with CI and normal pregnancies may not be the most suitable approach. Nevertheless, this type of comparison is still relevant considering that (1) there is a continuum of cervical competence, as shown by the increasing risk of SPTB with decreasing cervical length [7]; (2) CI may be considered as a non-measurable cervical length (i.e., 0 mm cervix on transvaginal sonographic assessment); and (3) the levels of inflammatory-associated mediators were inversely correlated with cervical length in women with a SCX (≤ 25 mm) [12, 13, 56]. Notably, in the present study, AF TSP2 was identified as a biomarker that could distinguish CI from SCX, despite no association between TSP2 and SPTB was found, suggesting that TSP2 may play a role in the process of cervical ripening and dilation. TSP2, a matricellular glycoprotein in the extracellular matrix, participates in cell-to-matrix communication and plays a significant role in the pathophysiological processes of collagen fibrillogenesis, angiogenesis, and wound healing by binding to pro-MMP-2 and MMP-2 [57, 58]. In agreement with the known biological role of TSP2 in the extracellular matrix, previous studies using pregnant mice showed that TSP2 deficiency is associated with premature softening of the uterine cervix [59, 60]. These studies further support the present finding that AF TSP2 levels are significantly higher in pregnancies complicated with CI than in those with SCX.

IPA analyses allowed the identification of important functional pathways that are differently involved in CI versus SCX, including granulocyte/agranulocyte adhesion and diapedesis; hepatic fibrosis/hepatic stellate cell activation; and cardiac hypertrophy signaling. Granulocyte/agranulocyte adhesion and diapedesis signaling pathway, as an important part of the innate immune response, is the first line of host defense against invading pathogens and is pivotal for recruiting granulocytes and agranulocytes to a site of infection or injury [61, 62]. The hepatic fibrosis/hepatic stellate cell activation pathway also represents a fundamental part of the wound healing in response to hepatic injury of any etiology (such as viral infection), which includes the characterization of matrix proteases and their inhibitors, cytokines, and apoptotic mediators [63, 64]. The cardiac hypertrophy signaling pathway is a relatively new concept, and thus its significance has not yet been fully defined. Nevertheless, a recent report suggests that inflammation, specifically the role of TLR4 signaling, plays an important role in the pathological mechanisms of cardiac hypertrophy [65]. Similar to our results, Govia *et al*. analyzed proteomic profiling of AF using normal pregnancy (genetic amniocentesis) as a control group and found chemotaxis, homing of leukocytes, activation of cells, and inflammatory response as the top activated biological functions associated with CI [66]. Taken together, these IPA data suggest that inflammatory and immune responses that have occurred in the uterine cavity may play an important role in the process of premature cervical dilation. Nonetheless, whether this inflammatory immune response is causative or resultant of premature cervical dilation remains unclear and needs to be further investigated.

The current study had several limitations. First, the study was retrospective in nature and the study participants were recruited over a period of 14 years to collect as extensive data as possible, despite advances in the management of CI/SCX during this period. Thus, it was not possible to apply the uniform standard of currently acceptable medical care to all patients, potentially modifying the impact of various AF proteins on SPTB risk. Second, the study did not include a control cohort of healthy pregnant women with normal cervical length in the same gestational age who underwent genetic amniocentesis and who delivered at term for determining a reference concentration range of the various evaluated proteins. Third, to measure protein concentrations in AF, frozen stored samples were used, potentially leading to alterations in the concentrations of the proteins measured. In fact, IL-6 and angiogenin concentrations in AF were reported to decrease over time despite storage under optimal freezing conditions [67]. Fourth, obtaining AF is an invasive procedure that has not been routinely used in clinical practice. Thus, the measurement of AF mediators studied may be clinically unfeasible for routine use. Fifth, owing to the small sample size in the subgroup analysis, there might be biased results toward the null hypothesis, especially in women with SCX. Additional studies with larger patient cohorts should be performed to further validate the reported findings. Finally, in the antibody microarray experiment, we conducted a nested case-control study for biomarker discovery using pooled samples from 20 CI and 20 SCX women. However, this approach may affect the selection of the DEPs and their related signaling pathways, according to previous studies on the effects of pooling samples on the performance of classification algorithms, which showed that misclassification rates are likely to increase with pool sizes in a linear pattern [68, 69]. Nonetheless, we believe that the pooled sample effect on our study is limited because a verification step was performed in which individual samples were analyzed in a relatively larger cohort for quantification of biomolecules. Moreover, the available literature does not provide the optimal number of samples to be used in pooled sample approaches for protein microarray experiments.

## Conclusions

Using protein–antibody microarray analysis of AF, several novel proteins and their specific signaling pathways were identified to be significantly altered between women with CI and SCX, most of which are involved in inflammation-immune and extracellular matrix remodeling. In addition, of the 15 potential differential mediators analyzed, elevated AF concentrations of EN-RAGE, S100A8/A9, and uPA were found to be independently associated with SPTB at < 32 weeks in patients with CI, whereas in patients with an SCX, increased AF levels of APRIL, EN-RAGE, LBP, and TNFR2 were related to SPTB. Further investigations are needed to confirm our results, and to determine whether these biomarkers could be useful to guide intervention strategies (such as cerclage) for CI and SCX to prevent harm and risks.

## Supporting information

S1 Table Demographic and clinical characteristics of the study population recruited for antibody microarray analysis [case-control study]. (DOCX)

S2 Table Proteins differentially expressed in amniotic fluid samples from women with cervical insufficiency as compared with those with a short cervix. (DOCX)

S3 Table Summary of the ingenuity pathway analysis of the 87 differentially expressed proteins in cervical insufficiency vs. a short cervix. (DOCX)

S4 Table Diagnostic indices of EN-RAGE, S100 A8/A9, and uPA in amniotic fluid to predict spontaneous preterm birth at < 32 weeks in women with cervical insufficiency (n = 80). (DOCX)

S5 Table Diagnostic indices of APRIL, EN-RAGE, LBP, and TNFR2 in amniotic fluid to predict spontaneous preterm birth at < 32 weeks in women with a short cervix (n = 49). (DOCX)

S1 File Raw data for the exploratory cohort. (SAV)

S2 File Raw data for the total cohort. (SAV)

S3 File (PDF)
