# Supplementary material for: Antibody microarray analysis of amniotic fluid proteomes in women with cervical insufficiency and short cervix, and their association with pregnancy latency length
Source: PLoS One. 2022 Feb 7;17(2):e0263586. doi: 10.1371/journal.pone.0263586 (PMC8820596; doi:10.1371/journal.pone.0263586)
Supplement: S1 Table — (DOCX) [file pone.0263586.s001.docx]

**S1** **Table.** Demographic and clinical characteristics of the study population recruited for antibody microarray analysis [case-control study]

| Characteristics | Cervical insufficiency  (n = 20) | Short cervix  (n = 20) | *P-*value |
| --- | --- | --- | --- |
| Age (years) | 32.6 ± 4.1 | 32.9 ± 4.2 | 0.620 |
| Nulliparity | 40.0% (8) | 50.0% (10) | 0.525 |
| Gestational age at sampling (weeks) | 21.6 ± 1.9 | 21.8 ± 1.9 | 0.758 |
| Cervical dilatation (cm)  ≥ 3 cm  < 3 cm | 2.0 (1.0-8.0)  45.0% (9)  55.0% (11) |  |  |
| Cervical length by ultrasound (mm) |  | 11.1 ± 4.5 |  |
| Positive amniotic fluid cultures | 0% (0) | 10 % (2) | 0.487 |
| Gestational age at delivery (weeks) | 37.5 ± 1.1 | 37.6 ± 0.9 | 0.758 |

Values are presented as mean ± standard deviation, median (range), or % (n).
