# Supplementary material for: Antibody microarray analysis of amniotic fluid proteomes in women with cervical insufficiency and short cervix, and their association with pregnancy latency length
Source: PLoS One. 2022 Feb 7;17(2):e0263586. doi: 10.1371/journal.pone.0263586 (PMC8820596; doi:10.1371/journal.pone.0263586)
Supplement: S2 Table — (DOCX) [file pone.0263586.s002.docx]

**S2 Table.** Proteins differentially expressed in amniotic fluid samples from women with cervical insufficiency as compared with those with a short cervix

| Identification of rectangles in figure 1 | Short name | Protein name | Fold-  change |
| --- | --- | --- | --- |
| 1 |  | Positive Control |  |
| 2 | Activin RIIA | Activin Receptor IIA | 1.37 |
| 3 | Adiponectin/Acrp30 | Adiponectin/Acrp30 | 1.42 |
| 4 | Angiopoietin-1 | Angiopoietin-1 | 3.49 |
| 5 | Angiopoietin-2 | Angiopoietin-2 | 2.02 |
| 6 | APRIL | A proliferation-inducing ligand | 0.60 |
| 7 | BD-1 | Beta-defensin 1 | 0.64 |
| 8 | BDNF | Brain derived neurotrophic factor | 0.72 |
| 9 | BIK | BCL2 interacting killer | 1.55 |
| 10 | CCR7 | C-C motif chemokine receptor 7 | 0.54 |
| 11 | CCR8 | C-C motif chemokine receptor 8 | 0.54 |
| 12 | CD14 | Monocyte differentiation antigen CD14 | 1.92 |
| 13 | CD40 Ligand/TNFSF5/CD154 | CD40 ligand | 2.80 |
| 14 | CD163 | Scavenger receptor cysteine-rich type 1 protein M130 | 1.56 |
| 15 | Chordin-Like 1 | Chordin-like 1 | 3.56 |
| 16 | Csk | C-terminal src kinase | 1.74 |
| 17 | CXCL14/BRAK | C-X-C motif chemokine ligand 14 | 0.45 |
| 18 | CXCR1/IL-8 RA | C-X-C motif chemokine receptor 1 | 0.65 |
| 19 | CXCR4 (fusin) | C-X-C motif chemokine receptor 4 | 1.88 |
| 20 | Dkk-3 | Dickkopf WNT signaling pathway inhibitor-3 | 10.57 |
| 21 | Endostatin | Endostatin | 1.80 |
| 22 | Erythropoietin | Erythropoietin | 1.90 |
| 23 | FGF-R4 | Fibroblast growth factor receptor-4 | 2.03 |
| 24 | FGF-9 | Fibroblast growth factor 9 | 1.43 |
| 25 | FGF-11 | Fibroblast growth factor 11 | 2.22 |
| 26 | FGF-13 1B | Fibroblast growth factor 13 1B | 1.57 |
| 27 | FGF-23 | Fibroblast growth factor 23 | 0.73 |
| 28 | Follistatin | Follistatin | 1.47 |
| 29 | GASP-1/WFIKKNRP | G-protein coupled receptor-associated sorting protein 1 | 1.93 |
| 30 | GDF3 | Growth differentiation factor 3 | 1.92 |
| 31 | GDF5 | Growth differentiation factor 5 | 1.87 |
| 32 | GDF-15 | Growth differentiation factor 15 | 0.39 |
| 33 | Glut5 | Glucose transporter 5 | 1.34 |
| 34 | Glypican 3 | Glypican 3 | 1.93 |
| 35 | HB-EGF | Heparin binding EGF-like growth factor | 1.47 |
| 36 | HRG1- α /NRG1- α | Neuregulin1 alpha | 0.38 |
| 37 | ICAM-1 | Intercellular adhesion molecule 1 | 0.58 |
| 38 | IGFBP-2 | Insulin like growth factor binding protein 2 | 0.68 |
| 39 | IGFBP-3 | Insulin like growth factor binding protein 3 | 0.62 |
| 40 | IL-1 R4/ST2 | Interleukin-1 receptor-like 1 | 0.31 |
| 41 | IL-8 | Interleukin 8 | 0.59 |
| 42 | IL-17 B | Inerleukin 17B | 2.56 |
| 43 | IL-17RC | Interleukin 17 Receptor C | 1.52 |
| 44 | IL-20 R β | Interleukin 20 Receptor beta | 0.27 |
| 45 | IL-22 | Interleukin 22 | 2.80 |
| 46 | IL-24 | Interleukin 24 | 2.63 |
| 47 | IL-28A | Interleukin 28A | 5.18 |
| 48 | IL-29 | Interleukin 29 | 1.34 |
| 49 | I-TAC/CXCL11 | Interferon-inducible T-cell alpha chemoattractant | 0.67 |
| 50 | Kininostatin/kininogen | Kininostatin/kininogen | 0.47 |
| 51 | Kremen-2 | Kremen-2 | 0.67 |
| 52 | Lck | Lymphocyte protein tyrosine kinase | 0.04 |
| 53 | Latent TGF- β bp1 | Latent transforming growth factor beta binding protein 1 | 0.52 |
| 54 | LBP | Lipopolysaccharide binding protein | 1.71 |
| 55 | LECT2 | Leukocyte cell-derived chemotaxin 2 | 2.15 |
| 56 | Lefty-A | Left-right determination factor 2 | 1.41 |
| 57 | Leptin R | Leptin Receptor | 2.59 |
| 58 | LIF | Leukemia inhibitory factor | 2.50 |
| 59 | LIF R α | Leukemia inhibitory factor receptor alpha | 2.47 |
| 60 | Lipocalin-1 | Lipocalin-1 | 1.52 |
| 61 | Lipocalin-2 | Lipocalin-2 | 1.44 |
| 62 | MIP-1β | Macrophage inflammatory protein-1β | 1.83 |
| 63 | MIP 2 | Macrophage inflammatory protein 2 | 2.12 |
| 64 | MMP-2 | Matrix metallopeptidase 2 | 178.02 |
| 65 | MMP-7 | Matrix metallopeptidase 7 | 0.47 |
| 66 | MMP-8 | Matrix metallopeptidase 8 | 2.63 |
| 67 | MMP-9 | Matrix metallopeptidase 9 | 3.21 |
| 68 | MMP-10 | Matrix metallopeptidase10 | 1.48 |
| 69 | NGF R | Nerve growth factor receptor | 1.53 |
| 70 | NRG3 | Neuregulin 3 | 1.34 |
| 71 | OSM | Oncostatin M | 1.35 |
| 72 | Progranulin | Progranulin | 1.32 |
| 73 | P-selectin | P-selectin | 1.68 |
| 74 | ROBO4 | Roundabout guidance receptor 4 | 1.45 |
| 75 | S100 A8/A9 | S100 calcium binding protein A8/A9 complex | 2.69 |
| 76 | SIGIRR | Single Ig IL-1-related receptor | 0.69 |
| 77 | Siglec-9 | Sialic acid binding Ig like lectin 9 | 0.76 |
| 78 | SPARC | Secreted protein acidic and rich in cysteine | 2.86 |
| 79 | Thrombospondin-1 | Thrombospondin-1 | 0.75 |
| 80 | Thrombospondin-2 | Thrombospondin-2 | 0.53 |
| 81 | TMEFF1/Tomoregulin-1 | Tomoregulin-1 | 1.46 |
| 82 | TNF Rl/TNFRSF1A | Tumor necrosis factor receptor I | 1.78 |
| 83 | TNF Rll/TNFRSF1B | Tumor necrosis factor receptor II | 5.03 |
| 84 | TSG-6 | TNF-stimulated gene 6 | 11.56 |
| 85 | Ubiquitin+1 | Ubiquitin+1 | 2.64 |
| 86 | uPA | Urokinase-type plasminogen activator | 2.24 |
| 87 | VEGF | Vascular endothelial growth factor | 2.74 |

Differential expression was determined by naked eye observation and ≥1.3-fold change (or ≤0.77-fold change) as threshold.
