# Supplementary material for: Antibody microarray analysis of amniotic fluid proteomes in women with cervical insufficiency and short cervix, and their association with pregnancy latency length
Source: PLoS One. 2022 Feb 7;17(2):e0263586. doi: 10.1371/journal.pone.0263586 (PMC8820596; doi:10.1371/journal.pone.0263586)
Supplement: S3 Table — (DOCX) [file pone.0263586.s003.docx]

**S3 Table.** Summary of the ingenuity pathway analysis of the 87 differentially expressed proteins in cervical insufficiency vs. a short cervix

| **Diseases and disorders** | *P*-value^†^ | | No.^‡^ |
| --- | --- | --- | --- |
| Connective tissue disorders | 1.32E-04 - 5.94E-21 | | 45 |
| Inflammatory disease | 1.32E-04 - 5.94E-21 | | 58 |
| Organismal injury and abnormalities | 1.32E-04 - 5.94E-21 | | 84 |
| Skeletal and muscular disorders | 8.27E-05 - 5.94E-21 | | 52 |
| Immunological disease | 1.04E-04 - 1.43E-20 | | 52 |
| **Molecular and cellular functions** | *P*-value^†^ | | No.^‡^ |
| Cellular movement | 1.29E-04 - 7.26E-24 | | 53 |
| Cell death and survival | 1.29E-04 - 3.33E-23 | | 56 |
| Cellular development | 1.07E-04 - 4.28E-19 | | 60 |
| Cellular growth and proliferation | 1.29E-04 - 4.28E-19 | | 59 |
| Cell signaling | 1.14E-04 - 5.26E-18 | | 46 |
| **Associated network functions** |  | | Score |
| Cellular movement, hematological system development and  function, immune cell trafficking |  | | 43 |
| Cell signaling, cell-to-cell signaling and  interaction, organismal development |  | | 38 |
| Cellular movement, hematological system development and  function, immune cell trafficking |  | | 33 |
| Cellular movement, cell death and survival, cell-to-cell signaling and interaction |  | | 14 |
| Inflammatory response, connective tissue disorders, inflammatory disease |  | | 10 |
| **Top canonical pathways** | *P*-value^†^ | | Ratio |
| Granulocyte adhesion and diapedesis | 6.60E-18 | | 16/165 (0.097) |
| Hepatic fibrosis/hepatic stellate cell  activation | 9.03E-16 | | 15/182 (0.082) |
| Agranulocyte adhesion and diapedesis | 1.46E-14 | | 14/176 (0.080) |
| Cardiac hypertrophy signaling (enhanced) | 5.85E-14 | | 19/476 (0.040) |
| Bladder cancer signaling | 2.00E-13 | 11/96 (0.115) | |

^†^ *P*-values are displayed in E notation: aEb indicates a value of a × 10^b^.

^‡^ Numbers of molecules involved.
