# Supplementary material for: Antibody microarray analysis of amniotic fluid proteomes in women with cervical insufficiency and short cervix, and their association with pregnancy latency length
Source: PLoS One. 2022 Feb 7;17(2):e0263586. doi: 10.1371/journal.pone.0263586 (PMC8820596; doi:10.1371/journal.pone.0263586)
Supplement: S4 Table — (DOCX) [file pone.0263586.s004.docx]

**S4 Table.** Diagnostic indices of EN-RAGE, S100 A8/A9, and uPA in amniotic fluid to predict spontaneous preterm birth at < 32 weeks in women with cervical insufficiency (n=80)

| Variables | Area (± SE) under the ROC curve | 95% CI | Cut-off value^a^ | Sensitivity^b^  (95% CI) | Specificity^b^  (95% CI) | PPV | NPV |
| --- | --- | --- | --- | --- | --- | --- | --- |
| AF EN-RAGE (ng/mL) | 0.744 ± 0.057 | 0.633– 0.856 | ≥ 9.07 | 90.9 (78.3–97.5) | 52.8 (35.5–69.6) | 70.2 | 82.6 |
| AF S100 A8/A9 (ng/mL) | 0.763 ± 0.054 | 0.656 – 0.869 | ≥ 2694.72 | 63.6 (47.8-77.6) | 83.3 (67.2-93.6) | 82.4 | 65.2 |
| AF uPA (ng/mL) | 0.649 ± 0.062 | 0.527 – 0.770 | ≥ 0.248 | 69.8 (53.9-82.8) | 58.3 (35.5-69.6) | 63.8 | 59.4 |

SE, standard error; ROC, receiver operating characteristics; CI, confidence interval; PPV, positive predictive value; NPV, negative predictive value; AF, amniotic fluid; EN-RAGE (S100A12), extracellular newly identified receptor for advanced glycation end products binding protein; S100 A8/A9, S100 calcium-binding protein A8/A9 complex; uPA, urokinase-type plasminogen activator.

^a^ Cut-off values corresponding to the highest sum of sensitivity and specificity.

^b^ Values are presented as % (95% CI).
