# Supplementary material for: Antibody microarray analysis of amniotic fluid proteomes in women with cervical insufficiency and short cervix, and their association with pregnancy latency length
Source: PLoS One. 2022 Feb 7;17(2):e0263586. doi: 10.1371/journal.pone.0263586 (PMC8820596; doi:10.1371/journal.pone.0263586)
Supplement: S5 Table — (DOCX) [file pone.0263586.s005.docx]

**S5 Table.** Diagnostic indices of APRIL, EN-RAGE, LBP, and TNFR2 in amniotic fluid to predict spontaneous preterm birth at < 32 weeks in women with a short cervix (n=49)

| Variables | Area (± SE) under the ROC curve | 95% CI | Cut-off value^a^ | Sensitivity^b^  (95% CI) | Specificity^b^  (95% CI) | PPV | NPV |
| --- | --- | --- | --- | --- | --- | --- | --- |
| AF APRIL (ng/mL) | 0.856 ± 0.060 | 0.738–0.975 | ≥ 0.473 | 100.0 (69.2–100) | 56.4 (39.7–72.2) | 37.0 | 100 |
| AF EN-RAGE (ng/mL) | 0.705 ± 0.114 | 0.482– 0.928 | ≥ 11.45 | 60.0 (26.2–87.8) | 89.7 (75.8–97.1) | 60.0 | 89.7 |
| AF LBP (ng/mL) | 0.756 ± 0.093 | 0.574 – 0.938 | ≥ 534.75 | 80.0 (44.4-97.5) | 64.1 (47.2-78.8) | 36.4 | 92.6 |
| AF TNFR2 (ng/mL) | 0.715 ± 0.118 | 0.484 – 0.947 | ≥ 10.77 | 70.0 (34.8-93.3) | 82.1 (66.5-92.5) | 50.0 | 91.4 |

SE, standard error; ROC, receiver operating characteristics; CI, confidence interval; PPV, positive predictive value; NPV, negative predictive value; AF, amniotic fluid; APRIL (TNFSF13), a proliferation-inducing ligand; *EN-RAGE* (S100A12), extracellular newly identified receptor for advanced glycation end products binding protein; LBP, lipopolysaccharide binding protein; TNFR2, tumor necrosis factor receptor 2.

^a^ Cut-off values corresponding to the highest sum of sensitivity and specificity.
