# Supplementary material for: Antibody microarray analysis of amniotic fluid proteomes in women with cervical insufficiency and short cervix, and their association with pregnancy latency length
Source: PLoS One. 2022 Feb 7;17(2):e0263586. doi: 10.1371/journal.pone.0263586 (PMC8820596; doi:10.1371/journal.pone.0263586)
Supplement: S3 File — (PDF) [file pone.0263586.s008.pdf]

- Supplementary Materials -

Membrane-based human antibody array

Protein levels of each of 40 discovery cohort samples were determined by bicinchoninic acid (BCA) assay (Micro BCA Protein Assay Kit, Thermo Fisher Scientific, Bremen, Germany). Equal amounts (25 μg) of protein from the individual AF samples were pooled into case (cervical insufficiency, n = 20) and control (a short cervix, n = 20) samples of 500 μg of protein each. The groups' relative expression levels of immunoregulatory proteins were profiled using a human antibody array kit (AAH-BLM-1B-2; RayBiotech, Norcross, GA) that has the capacity to simultaneously detect 507 human proteins, including cytokines, chemokines, matrix metalloproteases, adhesion molecules, growth and angiogenic factors, and adipokines. The AF samples from 20 women from each group (500 μg per group) were mixed and assayed in duplicate according to the manufacturer's suggested protocol. The membranes were exposed to a radiographic film (Kodak Industrex Processor Model; Eastman Kodak Company, Rochester, NY, USA), and the signal was detected and quantified using chemiluminescence image analysis (Bio-Rad Quantity 4.6.7; Bio-Rad Laboratories, Inc., Hercules, CA, USA) and the QuantityOne software (Bio-Rad ChemiDoc XRS Systems; Bio-Rad Laboratories, Inc.). The densities were exported to a Microsoft Excel spreadsheet (Microsoft, Redmond, WA, USA). The background intensity was subtracted from each image before analysis, and the signal intensities of each spot were normalized as a percentage of the positive controls on each membrane. To identify target proteins showing significant differences in signal intensity between case and control groups upon chemiluminescence

image analysis, the following spot selection criteria were applied simultaneously: (1) fold change (FC) of ≥1.3 or ≤0.77 for up- or downregulated proteins and (2) visible to the naked eye.

Analysis of various proteins in the amniotic fluid

The ranges of APRIL, DKK3, endostatin, EN-RAGE, IGFBP-2, IL-8, LBP, lipocalin-2, MMP-2, MMP-9, S100 A8/A9, SPARC, thrombospondin-2, TNFR2, and uPA standard curves were 31.2-2000 pg/mL, 31.2-2000 pg/mL, 62.5-4000 pg/mL, 7.8-500 pg/mL, 62.5-4000 pg/mL, 31.2-2000 pg/mL, 0.8-50 ng/mL, 78.1-5000 pg/mL, 0.7-20 ng/mL, 31.2-2000 pg/mL, 93.8-6000 pg/mL, 0.8-50 ng/mL, 156-10000 pg/mL, 7.8-500 pg/mL, and 62.5-4000 pg/mL, respectively. Prior to measurement of these proteins, the AF samples were diluted at 1:4 for APRIL and uPA, 1:10 for IL-8, MMP-2, and MMP-9, 1:100 for endostatin, EN-RAGE, LBP, SPARC, thrombospondin-2, and TNFR2, and 1:500 for DKK3, IGFBP-2, lipocalin-2, and S100 A8/A9. The intra- and interassay coefficients of variation were 7.8% and 10.5% for APRIL, 8.3% and 12.8% for Dkk-3, 1.9% and 6.1% for endostatin, 8.5% and 5.2% for EN-RAGE, 2.1% and 3.2% for IGFBP-2, 5.9% and 7.2% for IL-8, 3.3% and 10.9% for LBP, 8.7% and 10.0% for lipocalin-2, 2.4% and 2.1% for MMP-2, 5.2% and 1.5% for MMP-9, 3.2% and 3.0% for S100 A8/A9, 3.1% and 4.1% for SPARC, 2.8% and 4.5% for thrombospondin-2, 2.8% and 4.9% for TNFR2, and 5.5% and 4.0% for uPA, respectively.

Management of cervical insufficiency and an asymptomatic short cervix

Emergency cerclage was offered to patients with cervical insufficiency and performed with

the McDonald technique under spinal anesthesia. Prophylactic broad-spectrum antibiotics were administered in the operating room and after surgery in all women with cervical insufficiency. Tocolytics (magnesium sulfate, ritodrine or atosiban) were used at the discretion of the attending obstetrician when regular uterine contractions had developed. Patients who chose expectant management instead of cerclage were also given similar multifactorial treatments during their hospitalization, including prophylactic broad-spectrum antibiotics and tocolytic drugs at the discretion of their obstetricians. Decisions on the treatment for a short cervix, such as progesterone supplementation, placement of a cervical cerclage, and antibiotic treatment, were made at the discretion of attending obstetrician.

Maternal or fetal health status was carefully monitored for the development of clinical sign of chorioamnionitis and/or fetal compromise, both of which are indications for induction of labor. In most patients with culture-proven microbial invasion of amniotic cavity, labor was not induced or an elective cesarean delivery was not performed purely for positive AF cultures. In women with a diagnosis of intra-amniotic infection (i.e., positive AF cultures) but without clinical chorioamnionitis, the effective antibiotics against isolated bacteria were administered and close monitoring for clinical sign of chorioamnionitis and/or fetal compromise was performed until 34 weeks of gestation. Induction of labor was performed after 34 weeks if clinical sign of chorioamnionitis, fetal compromise or labor had not developed. Cesarean delivery was performed for standard obstetric indications at the discretion of the attending obstetrician.

Acute histologic chorioamnionitis was diagnosed when acute inflammatory change was detected in any tissue sample (umbilical cord, chorionic plate, chorion-decidua, or amnion), in accordance with previously published criteria.[1] Clinical chorioamnionitis was

diagnosed following the criteria proposed by Gibbs et al.[2]; fever (≥37.8℃) and the presence of two or more of the associated clinical findings (uterine tenderness, malodorous vaginal discharge, maternal leukocytosis, maternal tachycardia, and fetal tachycardia).

Supplementary References

1. Jung EY, Choi BY, Rhee J, Park J, Cho SH, Park KH. Relation between amniotic fluid infection or cytokine levels and hearing screen failure in infants at 32 wk gestation or less. Pediatr Res. 2017;81(2):349-355.

2. Gibbs RS, Blanco JD, St Clair PJ, Castaneda YS. Quantitative bacteriology of amniotic fluid from women with clinical intraamniotic infection at term. The Journal of infectious diseases 1982; 145(1): 1-8.
